# A Review of Von Hippel-Lindau Syndrome

**DOI:** 10.15586/jkcvhl.2017.88

**Published:** 2017-08-02

**Authors:** Neha Varshney, Amanuel A. Kebede, Harry Owusu-Dapaah, Jason Lather, Manu Kaushik, Jasneet S. Bhullar

**Affiliations:** 1 Department of Pathology, University of Toledo Medical Center, Toledo, OH, USA; 2 Department of Radiology, University of Toledo Medical Center, Toledo, OH, USA; 3 Department of Surgery, Providence Hospital and Medical Center, Southfield, MI, USA; 4 Department of Surgery, University of Minnesota, Minneapolis, St. Paul, MN, USA

**Keywords:** endolymphatic sac tumors, hemangioblastomas, pancreatic neuroendocrine tumors, pheochromocytoma, von Hippel-Lindau syndrome

## Abstract

Von Hippel-Lindau syndrome (VHL) is a familial neoplastic condition seen in approximately 1 in 36,000 live births. It is caused by germline mutations of the tumor suppressor gene VHL, located on the short arm of chromosome 3. While the majority of the affected individuals have a positive family history, up to 20% of cases arise from *de novo* mutations. VHL syndrome is characterized by the presence of benign and malignant tumors affecting the central nervous system, kidneys, adrenals, pancreas, and reproductive organs. Common manifestations include hemangioblastomas of the brain, spinal cord, and retina; pheochromocytoma and paraganglioma; renal cell carcinoma; pancreatic cysts and neuroendocrine tumors; and endolymphatic sac tumors. Diagnosis of VHL is prompted by clinical suspicion and confirmed by molecular testing. Management of VHL patients is complex and multidisciplinary. Routine genetic testing and surveillance using various diagnostic techniques are used to help monitor disease progression and implement treatment options. Despite recent advances in clinical diagnosis and management, life expectancy for VHL patients remains low at 40–52 years. This article provides an overview of the major clinical, histological, and radiological findings, as well as treatment modalities.

## Introduction

Von Hippel-Lindau (VHL) syndrome is an autosomal-dominant, multi-organ, familial neoplastic syndrome, which is caused by genetic aberrations of the tumor suppressor gene VHL. Germline mutations of the VHL gene predispose affected individuals to the development of benign and malignant tumors in several systems including the central nervous system (CNS), and visceral organs such as the kidneys, pancreas, adrenals, and reproductive organs. The most common VHL-associated tumors are hemangioblastomas involving brain, spinal cord, and retina; clear cell renal cell carcinoma (RCC); pheochromocytomas and paragangliomas; and pancreatic neuroendocrine tumors (PNETs) (1). Other findings include endolymphatic sac tumors (ELSTs) and papillary cystadenomas of the epididymis and broad ligament. VHL affects 1 in 36,000 live births and its inheritance pattern occurs in an autosomal dominant fashion, with a penetrance of over 90% by the age of 65. Early manifestations of VHL syndrome occur in the second decade of life and nearly 50% of patients are symptomatic at presentation. Cerebellar hemangiomas are the most common presenting symptom of VHL (2). Life expectancy of VHL patients was previously between 40 and 52 years, an average of 59.4 years for males and 48.4 years for females (3). VHL-related mortality is commonly due to complications related to RCC and CNS tumors.

## A brief overview of genetic and molecular biology

VHL gene is a tumor suppressor gene, located on the short arm of chromosome 3. It encodes for pVHL, a tumor suppressor protein involved in cellular signaling pathways. There are two isoforms of pVHL: pVHL30 and pVHL19. The pVHL30 isoform is composed of 213 amino acids. The smaller isoform, pVHL19, results from alternate translation initiation site in the open reading frame of codon 54, resulting in protein made of 160 amino acids, lacking the first 53 residues (4). Both isoforms are important for tumor suppressor effects of the VHL gene. Inactivating gene deletions, frameshifts, and missense mutations of the gene have been implicated in VHL syndrome. While the majority of VHL cases are familial, 20% of VHL cases are due to sporadic mutation. Most patients with VHL syndrome inherit a germline mutation of the VHL gene from affected parents and a wild-type gene from the unaffected parent. In affected individuals, germline mutations of the gene are present in all cell types of the body (first hit); however, only those cell types with a somatic inactivation of the normal allele are prone to tumor formation (second hit) (5). Thus, VHL follows Knudson’s two-hit hypothesis of hereditary tumorigenesis as in retinoblastoma.

The VHL gene product, pVHL, plays an important role in the regulation of hypoxia pathways through the transcription factor hypoxia-inducible factor (HIF), a heterodimeric molecule composed of alpha and beta subunits. The pVHL protein forms part of the VCB-CUL2 complex which is involved in ubiquitin-mediated degradation of HIF, and the tumor suppressive effects of functional pVHL are believed to be exerted through degradation of HIF (6). In normoxic environment, VCB-CUL2 complex binds to the alpha subunit of HIF, polyubiquitinates it, and marks for degradation. However, hypoxic conditions or functional loss of pVHL leads to stabilizations of HIF-α molecules, leading to sustained expression of pro-tumorigenic molecules including vascular endothelial growth factor (VEGF), platelet-derived growth factor (PDGF), erythropoietin, and transforming growth factor alpha (TGF-a) (7, 8). Subsequently, the upregulated target factors lead to rapid proliferations, tumorigenesis, and angiogenesis. HIF-independent mechanisms of VHL-mediated tumorigenesis include regulation of extracellular matrix, apoptosis, transcription, and stabilizing of microtubules (9, 10).

Patients can have variable presentations; the diagnosis is often confirmed by positive family history and presence of one VHL-associated tumor. In cases with no known history of VHL, the presence of multiple tumors is needed for diagnosis since approximately 20% of cases are the result of *de novo* mutations. Specific genotype–phenotype correlations in affected families led to the classification of VHL subtypes into types 1 and 2 (Table 1), primarily based on the presence of pheochromocytoma (11). Type 1 disease has a very low risk of pheochromocytomas. Type 2 VHL is further categorized into type 2a (low risk of RCC), type 2b (high risk of RCC), and type 2c which only presents with pheochromocytomas (12). Management of VHL-associated tumors requires multidisciplinary approach, and advances in genetic testing have led to early diagnosis of the syndrome. Various screening guidelines are in place to help monitor the progress of the different manifestation of VHL since early interventions can help prevent adverse outcomes (Table 2). Inheritance of the VHL syndrome occurs in an autosomal dominant fashion, and more than 300 germline mutations have been found in familial VHL cases. Hence, patients present with a wide spectrum of manifestations in the CNS, kidneys, adrenal glands, pancreas, and reproductive organs. An overview of the clinical features of these manifestations and their management is presented below.

**Table 1:** Classification of Von Hippel-Lindau Syndrome

Type	Clinical findings	Mutations
Type 1 (decreased risk for PCC)	Retinal and CNS HB, RCC, pancreatic cysts, and neuroendocrine tumors	Truncating or missense mutations
Type 2 (increased risk for PCC)		
Type 2A (low risk of RCC)	PCC, retinal HB, CNS HB	Missense mutation
Type 2B (high risk of RCC)	PCC, RCC, Retinal HB, CNS HB, pancreatic cyst, and neuroendocrine tumors	
Type 2C	PCC only	

Adapted from Refs. (6) and (15). HB, hemangioblastoma; PCC, pheochromocytomas; RCC, renal cell carcinoma.

**Table 2:** Surveillance of VHL manifestations

Age	Screening
**Ages 1 to 4**	**Annually** Eye or retinal examination with indirect ophthalmoscopy Thorough clinical examination to look for signs of neurological disturbance, nystagmus, white pupil, and abnormalities in blood pressure, or hearing or vision
**Ages 5 to 15**	**Annually** Physical examination and neurological assessment for blood pressure (lying and standing), hearing issues, neurological disturbance, nystagmus, strabismus, white pupil, and other signs Eye or retinal examination with indirect ophthalmoscopy. Abdominal ultrasonography annually from 8 years or earlier if indicated. Test for plasma metanephrines or urinary metanephrines using 24-h urine test **Every 2 to 3 years** Complete audiology assessment If repeated ear infections, MRI with contrast of the internal auditory canal for a possible endolymphatic sac tumor (ELST)
**Ages 16 and above**	**Annually** Thorough physical examination Eye or retinal examination with indirect ophthalmoscopy Ultrasound and MRI scan of the abdomen with and without contrast to assess kidneys, pancreas, and adrenals at least every other year Test for plasma metanephrines or urinary metanephrines using 24-h urine test **Every 2 years** MRI with contrast of brain, cervical, thoracic, and lumbar spine to rule out both ELST and hemangioblastomas Audiology assessment
**During pregnancy**	Regular retinal checkup to anticipate potentially more rapid progression of lesions Test for pheochromcytoma in early, mid, and late pregnancy During the fourth month of pregnancy, MRI (without contrast). If known retinal, brain, or spinal lesions, consider cesarean section

Adapted from Refs. (8) and (19).

## Central nervous system hemangioblastomas

### General features

CNS hemangioblastomas are the most common tumors in VHL syndrome affecting 60–80% of all patients (Table 3) (1, 13, 14). On average, the age of presentation is 33. The tumors are benign but are a major cause of morbidity and mortality due to mass effect on nearby CNS structures. These hemangioblastomas can be found in the cerebellum (16–69%), brainstem (5–22%), spinal cord (13–53%), cauda equina (11%), or supratentorial region (1–7%) (5, 14). Due to tumor growth, cysts and/or subsequent edema formation, the mass effect occurs within the CNS contributing to the clinical symptoms (5, 14, 15). Tumors can exhibit linear growth, exponential growth, or periods of fluctuation (14). When observed for more than 5 years, over 50% of hemangioblastomas will increase in size (16, 17). Any given patient may have various tumors that uniquely grow at different rates and patterns. High tumor burden is linked to partial germline mutation and male sex (5, 14).

**Table 3:** Frequency of lesions and average age range of presentation in VHL patients

Clinical Feature	Average (range) of presentation (years)	Frequency (%)	Reference
CNS hemangioblastoma	30 (9–78)	60–80%	1
Retinal hemangioblastoma	25 (1–67)	49–62%	4, 6
Endolymphatic sac tumors	31 (12–50)	6–15%	7, 15
Renal cell carcinoma or cysts	39 (16–67)	30–70%	6
Pheochromocytoma	30 (5–58)	10–20%	6
Pancreatic neuroendocrine tumors or cysts	36 (1–70)	35–70%	8, 15
Epididymal cystadenomas	Unknown (16–40)	25–60%	6, 15
Broad ligament cystadenomas	Unknown (16–46)	Unknown	8

### Radiological and histological findings

**Figure 1:**
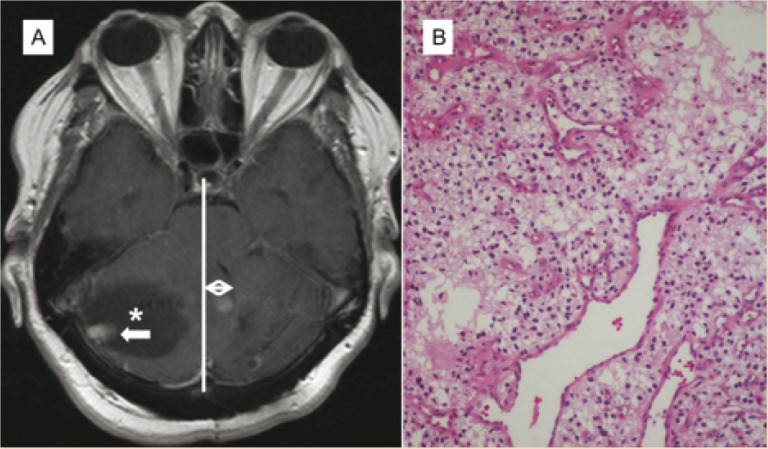
(A) Axial T1 postcontrast images demonstrate an enhancing mural nodule (white arrow) with accompanying cyst (*) in the right cerebellar hemisphere exerting a mass effect upon the midline and fourth ventricle (double white arrow). (B) Hematoxylin-eosin staining showing scattered large hyperchromatic nuclei, vacuolated cells, and multiple capillaries which are classic features of the cellular type of hemangioblastoma.

The modality of choice for detecting CNS hemangioblastomas is contrast-enhanced magnetic resonance (MR) imaging (5, 6, 14–18). Lesions as small as 2 mm will appear as enhancing lesions on T1-weighted images. On gross appearance, hemangioblastomas can be described as red vascular masses that reside within a layer of the thin capsule. The tumors are comprised of polygonal-shaped stromal cells flanked with several vascular structures (5). The blood vessels that supply larger hemangioblastomas are derived from migrating endothelial cells from nearby normal tissue. The polygonal stromal cells originate from multipotent hemangioblasts, which also differentiate into *de novo* tumor blood vessels (4) (Figure 1).

### Management

Craniospinal lesions are surgically resected safely and are often curative. Since CNS hemangioblastomas can grow at different sites and at irregular times, the approach to resect is withheld until patients are symptomatic (17). Asymptomatic lesions are monitored with annual imaging. Preoperative arterial embolization is indicated for extensive tumors to control tumor vascularity (19). Stereotactic radiosurgery (SRS) has been used to control solid, small CNS lesions (<3 cm diameter) in inoperable cases to prevent complications associated with microsurgery. Some studies report favorable short-term tumor control rates with SRS; however, there is no long-term benefits as SRS-treated tumors continue to grow at similar rates compared to the natural progression of the hemangioblastomas (20). Future studies are needed to assess the potential long-term effects and benefits of SRS.

## Retinal hemangioblastomas

### General features

Retinal hemangioblastomas arise frequently in VHL patients. They are seen in as many as 60% of patients (Table 3) (7, 14). Although not the most common tumor in VHL, they are often the first manifestation. The median age of onset is 21, which is the lowest among the other clinical features (5, 14). These retinal lesions are symptomatic at presentation. All patients who present with one retinal hemangioblastoma will likely develop bilateral lesions by age 56 (14). Complications of retinal hemangioblastomas include blindness or severe visual deficits in 5–8% of VHL patients (7, 14). The disease may occur at the periphery or optic nerve. Both can lead to symptoms as a result of the accumulation of subretinal fluid and development of hard exudates in the macula. Worse visual outcomes are associated with early age of onset of ocular disease, bilateral involvement, and missense mutations in VHL (7, 14, 17).

### Radiological and histological findings

Retinal hemangioblastomas resemble CNS hemangioblastomas grossly and histologically. The level of gliosis and hemorrhage may indicate the severity of the lesion. The ophthalmoscope in conjunction with pharmacological dilation of the iris allows for the identification of most retinal tumors. Fluorescein angiography is used to evaluate macular function associated with new lesions in the periphery or optic nerve. Screening exams should be conducted annually beginning at age 1, with dilated fundoscopy (7).

### Management

Management of capillary retinal hemangioblastomas includes laser photocoagulation, cryotherapy, and diathermy depending on the location and size of the lesions. Photocoagulation is used for controlling small capillary hemangiomas growing in the posterior part of the retina and has been shown to be very effective in treating tumors that are 1.5 mm or smaller (7). Response rates to direct laser coagulation have been reported to be more than 90%. Cryotherapy is indicated in managing retinal hemangiomas located anteriorly and is most effective in tumors with less than 3 mm in diameter. Vitreoretinal surgery is utilized in cases where retinal detachment due to traction occurs and exudation is present. To prevent blindness, hemangioblastomas near the optic nerve are treated with intravitreal anti-VEFG therapy to control growth and help ameliorate edema and exudate formation (14). External beam radiotherapy can be performed in the treatment of refractory cases.

## Endolymphatic sac tumors

### General features

ELSTs develop from endolymphatic epithelium within the vestibular aqueduct (16). The endolymphatic system plays a role in the production and resorption of endolymph which is found within the cochlea and semicircular canals. These tumors are found in 6–15% of VHL patients; however, they are rare in the general population (Table 3) (6, 14). They generally appear around 31 years of age. They are benign tumors that do not metastasize but may be locally aggressive (6). Patients may present with ear fullness, disequilibrium, and hearing loss. Larger lesions (>3 cm) can disturb the facial nerve resulting in facial paresis (14, 16).

### Radiological and histological findings

Diagnosis of ELSTs through radiologic examination should include the use of precontrast and postcontrast computed tomography (CT) and magnetic resonance imaging (MRI) (5, 6, 14). The internal auditory canal is the target location. On CT, these tumors exhibit similar intensity as the brain parenchyma; however, there may be focal areas of low and high attenuation. Centered within the endolymphatic duct, large tumors are visualized as expanding into the temporal bones on CT (5). On precontrast T1-weighted MRI, the level of intensity may be either homogeneous or heterogeneous. Postcontrast T1-weighted MRI will show variable patterns of enhancement or homogeneity. To aid the assessment of hearing loss, audiograms are employed to supplement CT and/or MRI imaging (16). ELSTs are richly vascular, often eroding the nearby temporal bone. Histologically, these lesions are papillary cystic glandular neoplasms with variable patterns. Due to their association with local bone erosion and adenomatous appearance, these tumors were formerly referred to as low-grade adenocarcinomas (5, 14, 16). When endolymphatic tumors are small, they can be entirely found within the endolymphatic sac. Conversely, they invade and erode the temporal bone when they are large (16).

### Management

Surgical resection is the mainstay therapy for ELSTs that are detectable on radiographs in order to prevent sensorineural hearing loss and treat audiovestibular symptoms. Complete resection is also indicated for patients with an evidence of intralabyrinthine hemorrhage, but no visible tumors on imaging studies as hemorrhage indicate presence of microscopic ELSTs (15). Most patients who undergo total resection of tumors retain their hearing ability. Rates of recurrence are minimal in the majority of cases, approximately 3% (21). Radiation therapy may have a role in unresectable tumors; however, not enough studies have been conducted to assess its efficacy.

## Renal cell carcinoma and renal cysts

### General features

Renal manifestations of VHL include benign renal cysts and clear cell carcinoma (1, 6). Multiple bilateral renal cysts are found on screening in 50–70% of VHL patients (Table 3) (14). RCC may affect up to 30% of VHL patients. They are rarely the first sign of VHL. Renal cysts are asymptomatic, and unlike autosomal dominant polycystic kidney disease, the incidence of chronic renal failure is low. Bilateral RCCs and renal cysts present in the third or fourth decade of life in these patients (22). Up to 70% of VHL patients develop RCC by 60 years of age; RCC is the leading cause of mortality in this group of patients (14). VHL renal lesions range from simple cysts to entirely solid lesions (6). Clinical findings include renal mass with flank pain or hematuria (1, 6, 14). Simple renal cysts are generally asymptomatic, while complex cysts may become solid renal masses. Renal function can be preserved despite having multiple cysts (16).

### Radiological and histological findings

**Figure 2:**
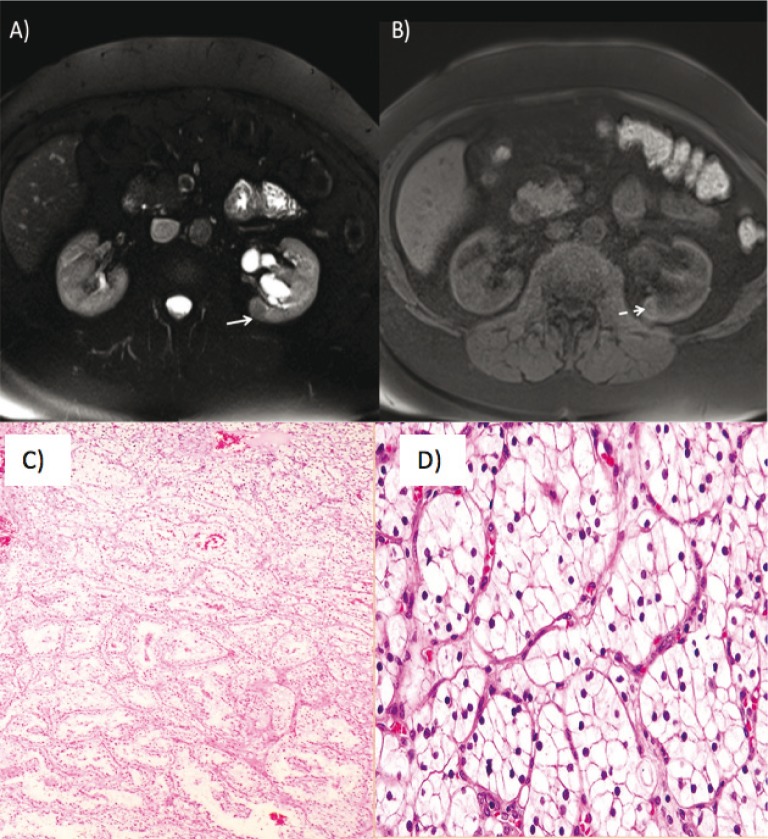
(A) Axial T2 weighted images show a T2 hypointense lesion in the posteromedial aspect of the midportion of the right kidney (white arrow). (B) Axial T1 postcontrast images show enhancement of the lesion (dashed white arrow) indicating a solid renal mass such as renal cell carcinoma. (C) Low-power- and (D) high-power-hematoxylin-eosin staining showing a typical picture of clear cell renal cell carcinoma with nests of clear cells surrounded by intricately branching vascular septa.

Serial imaging of the kidneys is useful in detecting RCC because it often remains asymptomatic for long periods of time (5, 6, 14, 16). Simple renal cysts in VHL syndrome are usually asymptomatic and rarely need intervention. More complex cysts require monitoring as they may have some solid components of RCC. Diagnosis during the presymptomatic stage has the potential to improve outcomes. Abdominal CT with contrast is the gold standard for diagnosis of renal lesions of VHL (5, 14). It allows clinicians to quantify the size and the number of tumors and cysts. CT can differentiate simple cysts from complex cysts (14). Abdominal MRI is also accurate in this regard. RCC and cysts in VHL are the clear cell type (5). VHL-related RCC is histologically similar to sporadic clear cell carcinoma. They often have low-grade histology (Figure 2).

### Management

RCCs are typically detected on routine screening, and no intervention is necessary for tumors less than 3 cm in diameter. Partial nephrectomy is the treatment of choice for tumors that have reached the 3 cm threshold to reduce the risk of metastasis while maintaining kidney function. Nephron sparing surgeries on lesions greater than 3 cm have 10-year cancer survival rates as high as 81% with preservation of renal function (14). Percutaneous and laparoscopic radiofrequency ablation therapy has been proven to be effective in treating smaller tumors (<3 cm) with low complication rates (23). However, lesions ablated with radiofrequency need frequent monitoring and intervention.

## Pheochromocytomas

### General features

Pheochromocytomas occur in up to 20% of VHL patients (Table 3). The hereditary syndromes, subtypes, susceptibility genes, and sites of pheochromocytomas are summarized in Table 4. They can be bilateral and occasionally multifocal. Pheochromocytomas usually present in the second decade of life in VHL patients and rarely transform into malignant tumors. These tumors produce catecholamines, such as excessive norepinephrine, causing hypertension, tachycardia, palpitations, headaches, sweating, pallor, and nausea (5, 13).

**Table 4:** Hereditary syndromes and subtypes of pheochromocytoma

Syndrome	Susceptibility gene	Tumor location	Reference
MEN type 2A, 2B	RET	Adrenal (bilateral)	26
VHL type 2A, 2B, 2C	VHL	Adrenal (bilateral)	4
Neurofibromatosis type 1	NF1	Adrenal	26
Familial paraganglioma syndrome type 1, 3, 4	SDHA, SDHB, SDHC	Head and neck, adrenal, extra-adrenal (i.e., gastrointestinal stromal tumor)	14, 26
Familial pheochromocytoma	Chr 2, 16	Adrenal, extra-adrenal	26

### Radiological and histological findings

**Figure 3:**
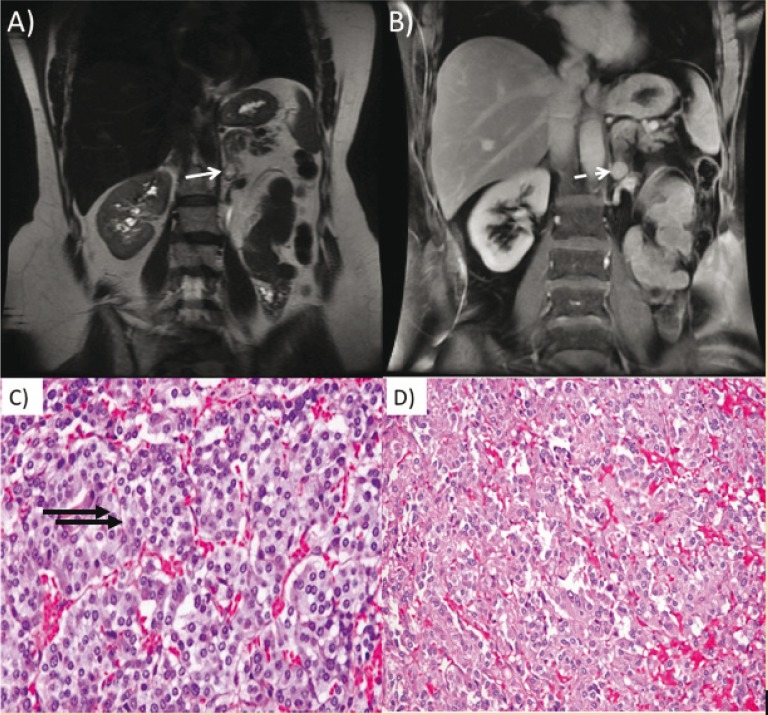
(A) Coronal Single-shot fast spin echo (SSFSE) image of the abdomen shows a high signal lesion (white arrow) within the medial limb of the left adrenal gland. (B) Coronal T1 postcontrast image shows a homogeneously enhancing mass (dashed white arrow) consistent with pheochromocytoma. (C) and (D) Hematoxylin-eosin staining showing nests of tumor cells (zellballen growth pattern) surrounded by a discontinuous layer of sustentacular cells and fibrovascular stroma intermixed with blood.

The diagnosis of pheochromocytoma is based on biochemical laboratory and imaging studies (5, 13, 14). Functional laboratory tests are useful especially when imaging does not show tangible evidence of a lesion. Plasma free metanephrines is the most sensitive method (97% sensitivity) for detecting pheochromocytoma more so than the 24-h urinary measurement of catecholamines which can yield false negative results (14). Postcontrast abdominal CT imaging is sensitive in detecting adrenal and extra-adrenal pheochromocytomas. Contrast-enhanced MRI of the abdomen is between 90% and 100% sensitive in the detection of these tumors and is the preferred modality compared to CT. Identification of extra-adrenal tumors may benefit from meta-iodobenzylguanidine (MIBG) scintigraphy (5, 6, 13, 14). On histology, classic pattern is seen as small nests (zellballen) of neuroendocrine cells (chief cells) with interspersed small blood vessels. However, numerous variant and combined patterns exist, including diffuse growth, large zellballen, spindle cells, cell cords, etc. (Figure 3).

### Management

The preferred treatment for pheochromocytomas is surgical resection with part adrenalectomy by laparoscopic approaches. Perioperative management of pheochromocytomas with a combination of alpha-adrenergic and beta-adrenergic blockade is necessary to prevent catecholamine-mediated life-threatening complications such as cardiac arrhythmias, hypertensive crisis, and myocardial infarction. Pharmacological intervention for 10–14 days is required prior to surgery. Early cortical-sparing partial adrenalectomy has shown low recurrence rates in a follow-up of 36 VHL patients with pheochromocytomas (24).

## Pancreatic neuroendocrine tumors and cysts

### General features

About 35–70% of patients with VHL present with pancreatic findings (Table 3). Up to 72% of VHL patients will have pancreatic cysts on autopsy (5, 6, 9–11, 13, 14). There are usually multiple cysts that present without symptoms. Serous cystadenomas and neuroendocrine tumors are other manifestations of pancreatic lesions in VHL syndrome (5). In 12% of patients, pancreatic cysts may be seen as the only sign of VHL (13, 14). Neuroendocrine tumors can become malignant and metastatic in 8% of patients (14). Due to the replacement of pancreatic parenchyma, cysts and cystadenomas of the organ can cause exocrine or endocrine deficiency (5, 6). Compression of nearby structures (e.g., the intestines or bile duct) can lead to symptoms (14, 16). Neuroendocrine tumors of the pancreas are rarely the cause of morbidity and mortality. However, tumor conversion to malignancy or metastasis may lead to a poor prognosis. High tumor burden is associated with size greater than 3 cm and growth that doubles in less than 500 days (14, 16).

### Radiological and histological findings

**Figure 4:**
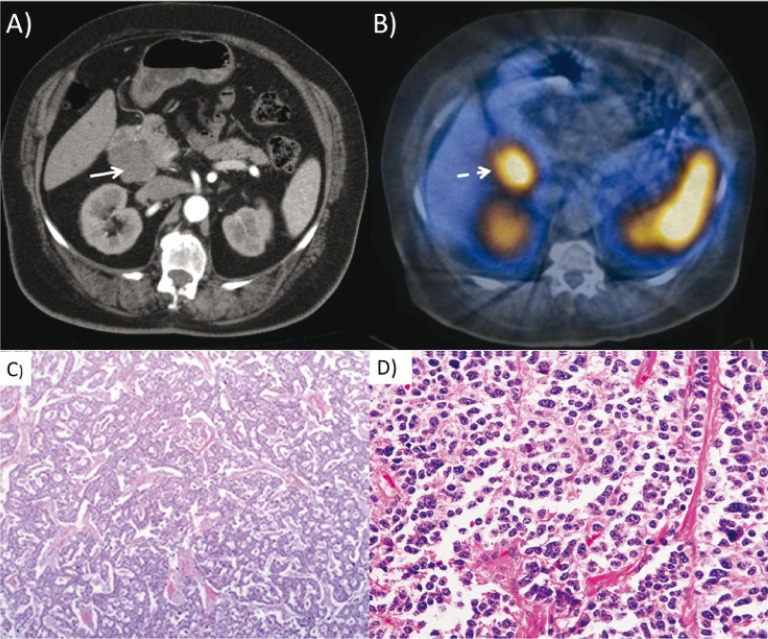
(A) Axial CT images of the abdomen with contrast show a mass in the head of the pancreas (white arrow) in a patient with abnormal gastrin levels clinically. (B) SPECT-CT images of a patient after administration of In-111-octreoscan show abnormal uptake in the pancreatic head mass (dashed white arrow). (C) Low-power- and (D) high-power-hematoxylin-eosin staining showing uniform neuroendocrine cells.

Pancreatic cysts are often clinically silent; therefore, routine imaging is important for diagnosis in VHL patients (14). On postcontrast CT imaging, a PNET can be seen as an enhancing mass (5, 6). Once the presence of the tumor is established, MRI can help confirm the diagnosis. Endoscopic ultrasound and somatostatin receptor scintigraphy are additional options for detecting these lesions (5, 14). FDG positron emission tomography (PET) has been utilized to identify neuroendocrine tumors that are not visible by CT. PNETs are well circumscribed and encapsulated in nature. From a histological standpoint, these tumors are derived from pancreatic islet cells. Originally, they were called islet cell tumors. Using immunohistochemistry, the neoplastic cells stain positive for pancreatic and gastrointestinal hormones (5). Pancreatic cysts and cystadenomas consist of clear glycogen-rich epithelial cells and fibrous stroma (Figure 4).

### Management

Pancreatic cysts do not require surgical intervention; however, surgical compression is indicated for patients with obstructive symptoms. PNETs with a potential for metastatic disease are resected with enucleation by Whipple’s procedure or partial pancreatectomy depending on the size and location of the tumor (5). Resection criteria involve tumor size greater than 3 cm, a pathogenic variant in exon 3, and tumor with a doubling growth rate in less than 500 days, all of which increases the risk of metastasis (25).

## Epididymal cystadenomas

### General features

Males with VHL can develop cystadenomas in the epididymis. They may arise unilaterally or bilaterally in 25–60% of VHL males (Table 3). They are benign in nature and do not require surgery (14, 16). These tumors typically appear during teenage years (16). They are often asymptomatic and incidentally detected. In 17% of patients, the lesions are unable to palpate due to a smaller size (14). Ultrasonography is the modality of choice in locating these lesions. The tumors share morphologic features of other VHL tumors (6, 14, 16).

### Radiological and histological findings

Diagnosis of epididymal cystadenomas is made by palpation and confirmed with ultrasound (5, 6, 14). These lesions can be difficult to palpate due to small size; therefore, ultrasonography is the modality of choice (14). The testes are a common site for RCC metastasis. On gross appearance, epididymal cystadenomas are predominantly solid but contain numerous cysts comprised of colloid material (5, 6, 8, 14). Histologically, the cysts are lined by clear epithelium.

### Management

Due to their benign nature, epididymal cystadenomas are managed conservatively and are routinely followed with physical exam and ultrasonography. Surgery is rarely performed to relieve compressive symptoms of these tumors.

## Broad ligament cystadenomas

### General features

Papillary cystadenomas are rare but found in the mesosalpinx and the broad ligament (5, 14, 16). They may be present unilaterally or bilaterally in women with VHL syndrome. The incidence is unknown (Table 3). The tumor may present as an abdominopelvic mass with symptoms of abdominal discomfort or a painful adnexal mass (5, 14, 16).

### Radiological and histological findings

Broad ligament masses may be detected by MRI of the abdomen or pelvic ultrasound. Histologically, papillary cystadenomas are similar to cystadenomas of the epididymis with respect to the prominent papillary architecture (6, 14, 16). The core of the lesions has a fibrovascular and hyaline stroma (5).

### Management

These tumors are also benign and asymptomatic; thus, they are managed conservatively. CT or ultrasonography is typically used to monitor their growth over time.

## Surveillance protocols

Surveillance is not only crucial in detecting new lesions at an early stage but also crucial for monitoring small asymptomatic lesions. Improved understanding of clinical features, better imaging techniques, and improvement in therapy have decreased morbidity and mortality in patients with VHL syndrome. Surveillance is most important in hemangioblastomas, RCCs, and pheochromocytomas as they most often result in severe disability or death. Table 2 summarizes the recommendations made by the VHL Alliance in conjunction with their Medical Advisory Board.

## Genetic counseling

For patients with at-risk pregnancies in which at least one of the parents has known VHL syndrome, genetic diagnosis and subsequent counseling should be made available for better understanding of the disease (26). Approximately 80% of patients diagnosed with VHL have one affected parent. About 20% of individuals are without a positive family history and have a *de novo* pathogenic variant. Genetic testing is recommended for parents of an individual/proband with a *de novo* pathogenic variant (19). If the pathogenic variant in the proband is not known, screening for retinal lesions and abdominal ultrasound should be offered to the parents at the very minimum. Family history may appear to be negative for a number of reasons, such as the early death of a parent or reduced penetrance. Consequently, a purported negative family history cannot be confirmed without proper genetic counseling performed on both parents of the affected individual. The risk of VHL to other family members, such as the siblings of the proband, depends on the status of the proband’s parents. Among patients with somatic mosaicism, the risk to offspring depends on whether the germ tissue carried the mutation. Since it is difficult to determine clinically, patients with documented mosaicism should be counseled regarding the risk. Families should keep in mind that the best time to determine genetic risk of VHL and establish prenatal testing is before a pregnancy. Genetic counseling, which includes an explanation of potential risk to offspring and reproductive options, is appropriate for young adult patients who are affected or at risk.

## Conclusion

VHL is a multisystem cancer syndrome with common manifestations affecting the CNS and several visceral organs. Management of VHL syndrome is complex and requires input from various specialties. With advances in genetic testing, identification of mutations in affected individuals is readily accomplished, leading to an early intervention to reduce morbidity and mortality. Routine surveillance for common VHL manifestations is used to monitor disease progression and help guide treatment for tumors that can potentially metastasize. Surgical resection of hemangioblastomas in VHL has been shown to be effective with long-lasting outcomes. As VHL has many endocrine manifestations, patients require regular follow-ups and interventions after undergoing treatment. With advances in understanding the molecular biology of VHL syndrome, several therapies targeting downstream targets of VHL, such as VEGF, are currently available in treating patients with common manifestations such as RCC. The future challenge is to understand precise molecular changes and develop new therapies for the various manifestations of the syndrome.
